# A parallel evaluation of 5 indirect cost-effective methods for assessing failure of passive immunity transfer in neonatal calves

**DOI:** 10.3168/jdsc.2019-17931

**Published:** 2020-09-02

**Authors:** A. Mugnier, K. Pecceu, F. Schelcher, F. Corbiere

**Affiliations:** 1 UMR INRAE-ENVT 1225 IHAP, Ecole Nationale Vétérinaire, Université de Toulouse, 23 chemin des Capelles, F-31076 Toulouse Cedex 3, France; 2 MSD Santé Animale, 7 Rue Olivier de Serres, CS 17144, 49071 Beaucouzé, France

## Abstract

The aim of this study was to evaluate the diagnostic accuracy of 5 indirect methods that may be used in veterinary practices to assess the success of maternal immunoglobulins transfer in neonatal calves. Blood samples (n = 245) were collected from 2- to 6-d-old calves. Serum immunoglobulin concentrations were determined by radial immunodiffusion. Serum total protein and albumin concentrations and γ-glutamyl transferase activity were determined by veterinary practitioners using their own biochemistry analyzer. Globulin concentration was calculated. Veterinary practitioners were also asked to assess serum total protein using a digital Brix refractometer (%Brix) and an optical refractometer (g/L). Test sensitivity (Se) and specificity (Sp) were calculated using radial immunodiffusion as the reference standard, with failure of transfer of passive immunity defined as IgG <10.0 g/L. Receiver operating characteristics curves were created and optimal cutoff values for each of the 5 indirect measurement methods were selected based on the maximization of the Youden's *J* statistic. In this study, Globulin concentration yielded the highest accuracy to identify failure of transfer of passive immunity (Se = 89.4%; Sp = 89.3% at 34 g/L). For %Brix, the optimal combination of Se and Sp was at 8.4% Brix (Se = 86.5% and Sp = 83.8%). The use of an optical refractometer was associated with the lowest diagnostic accuracy (Se = 69.7% and Sp = 81.6% at 52 g/L). For γ-glutamyl transferase activity, the optimal cut-points were different for 2- to 3-d-old calves (Se = 87.5% and Sp = 87.8% at 393 IU/L) or 4- to 6-d-old calves (Se = 90.0% and Sp = 86.4% at 254 IU/L). Because it does not require any specialized laboratory equipment, digital Brix refractometer can be a valuable and inexpensive tool for on-farm use, to help producers and veterinary practitioners in their calf health management programs.

## Highlights

• Failure of transfer of passive immunity is frequent in beef and dairy neonatal calves.
• Assessing the transfer of maternal immunoglobulins is of major importance.
• All five indirect methods evaluated provide reasonably accurate results.
• The digital Brix refractometer is cost-effective and its use should be promoted.

## Graphical Abstract

**Figure:**
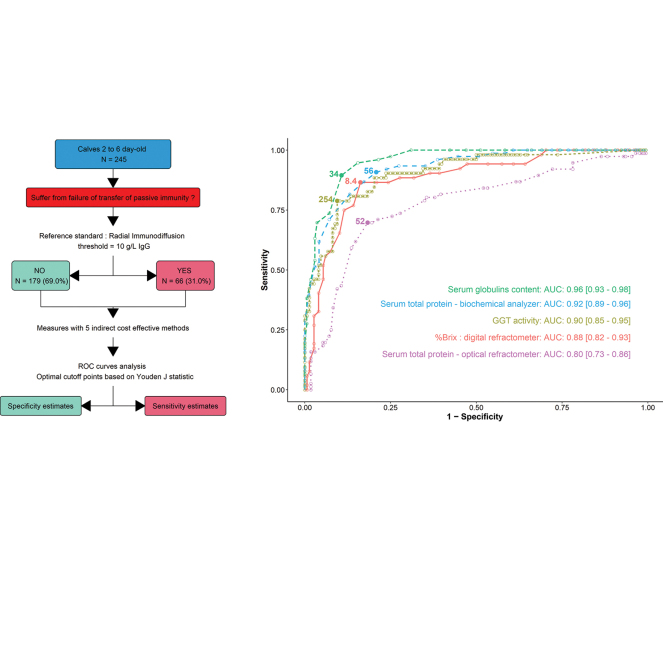
**Summary:** The aim of this study was to evaluate the diagnostic accuracy of 5 indirect methods to assess the transfer of maternal immunoglobulins in neonatal calves. Because it does not require any specialized laboratory equipment, a digital Brix refractometer is a valuable and inexpensive tool for on-farm use, to help producers and veterinary practitioners in their calf health management programs.

Newborn calves are agammaglobulinemic, and protection from infectious diseases mostly depends on the quality of passive immunity transfer from colostrum (Elsohaby et al., 2015). Failure of transfer of passive immunity (**FTPI**) is associated with increased risk of mortality, severe diarrhea, and respiratory diseases in neonatal calves (Donovan et al., 1998). Passive immunity status also influences longevity, future milk production, and weight gain (Vogels et al., 2013; Thornhill et al., 2015; Raboisson et al., 2016). FTPI is classically diagnosed when serum IgG concentration is <10.0 g/L (Deelen et al., 2014; Elsohaby et al., 2015; Buczinski et al., 2018), but the 8.0 g/L threshold has sometimes been used (Buczinski et al., 2018). Despite the fact that best practices regarding colostrum management have been widely spread in the scientific and technical literature, FTPI remains a major issue in many herds (Weaver et al., 2000; Godden, 2008). Depending on the country, farming system, and decision thresholds, 19 to 40% of calves may suffer from FTPI (Vogels et al., 2013; Elsohaby et al., 2015; Buczinski et al., 2018; Raboisson et al., 2016).

Several methods have been developed to assess the adequacy of colostral immunoglobulin transfer in the neonatal calf. The historical gold standard is a direct measurement of serum IgG concentration using radial immunodiffusion (**RID**); however, this method requires 18 to 24 h to get the results, is expensive, and cannot be used on a farm. There is a need for easier-to-use and cost-effective methods that can rapidly determine the immune status of neonatal calves. Direct or indirect assessment of serum total protein (**STP**) concentration is considered a good proxy of IgG serum concentration (Tyler et al., 1998; Hernandez et al., 2016; Elsohaby et al., 2019a). The diagnostic accuracy of these methods has mostly been evaluated in controlled environments (laboratory tests done by the same researcher), and field experiments remain rare. In the current study, 5 methods conducted by veterinary practitioners under field conditions were evaluated in parallel and compared: assessment of STP by biochemistry analyzer in veterinary practice, assessment of STP by optical refractometry, digital Brix refractometry, calculated serum globulin concentration (**GLOB**), and serum γ-glutamyl transferase (**GGT**) activity level.

For a desired absolute precision of ±10%, expected values for sensitivity (**Se**) and specificity (**Sp**) estimates of 80%, and a confidence level of 95%, the minimum required sample size was 62 (Manitz et al., 2017). Under the assumption that the FTPI prevalence would be around 25% in our study population, the minimal total sample size was 248. The present study included 258 beef and dairy calves from 93 herds, with 1 to 6 calves (median 3) sampled per herd. Calves were randomly selected from each herd, and sampling was performed during regular farm visits by veterinary practitioners.

Whole blood was collected from calves by jugular venipuncture into 2 vacuum tubes without anticoagulant. Blood samples were refrigerated for 24 h before serum was separated by centrifugation, with one aliquot being immediately frozen at −20°C. One serum aliquot was used by veterinary practitioners to get an indirect estimate of STP, using an optical refractometer (various trademarks, **STP_OP**, g/L) and a digital Brix refractometer was also applied (MA882, Milwaukee Instruments, Szeged, Hungary, **%Brix**). Veterinary practitioners used their VetTest biochemistry analyzers to estimate GGT (EC 2.3.2.2, IU/L) activity, STP (**STP_BA**, g/L), and serum albumin concentrations (**ALB**, g/L). Serum globulin concentration (g/L) was determined by subtracting ALB from STP_BA. All measures were performed by 15 veterinary practitioners, from 14 practices, who had previously been individually trained to use the optical and digital Brix refractometers.

The frozen serum samples were transported to ISAE 35 laboratory (Combourg, France), where they were maintained at −20°C until thawing for IgG concentration determination using a commercial RID assay (RID-IgG, g/L, Bov IgG Ring Test, ID Biotech, Issoire, France).

At sampling, 51.2% of calves (132/258) were 2 and 3 d old, 43.8% (113/258) were 4 to 6 d old, 1.2% (3/258) were 0 to 1 d old, and 1.2% were older than 6 d old (3/258). For the 7 remaining calves (7/258, 2.7%), the information was missing. Calves 0 and 1 d old, calves older than 6 d, and calves with missing age information were excluded, leaving 245 calves between 2 and 6 d old for the final analysis.

The Pearson product moment correlation coefficient (r) between each test and the RID-IgG concentration were calculated. Then, we evaluated the diagnostic characteristics of each indirect method for detecting FTPI (IgG <10.0 g/L), using RID-IgG concentration as the reference standard. The Se of an indirect method was defined as the conditional probability of correctly identifying animals with FTPI (IgG concentration <10.0 g/L FTPI+) and Sp as the conditional probability of correctly identifying animals without FTPI (IgG ≥10.0 g/L FTPI−).

Receiver operating characteristic (**ROC**) curves were used to identify the optimal cutoff point for each indirect method. Cutoff values were selected based on the maximization of Youden's *J* statistic (*J* = Se + Sp − 1) (Hajian-Tilaki, 2013). Statistical uncertainty was assessed by calculating exact 95% binomial confidence intervals (95% CI). The area under the ROC curve (**AUC**) and the distance between the curve and the upper left-hand corner of the ROC space (d^2^) were also calculated as an effective and combined measure of Se and Sp to estimate the ability of the test to discriminate between the classes (FTPI+ vs. FTPI−) (Hajian-Tilaki, 2013). Finally, Cohen's kappa coefficients (κ) were computed to show the level of agreement between the evaluated tests and the reference RID assay at the selected optimal cutoffs.

The precision and repeatability of the RID assay were assessed by intra-assay CV, based on 10 repeated measures for 5 independent serum samples with IgG concentrations ranging from 2 to 30 g/L. Intra-assay coefficients of variation were between 4.8% and 8.2% (mean 6.2%), and RID was deemed to be repeatable enough for the purpose of the study.

Statistical analyses were performed in R software (version 3.5.0) with ROC curves fitted with the pROC package (Robin et al., 2011).

Descriptive statistics for tests results are shown in Table 1. The mean serum RID-IgG concentration was 16.26 g/L (SD ± 8.91). Seventy-six samples (31.0%) had IgG concentrations <10.0 g/L, indicating FTPI. Due to missing values for some calves, sample sizes were different from one test to the other. Results based on the sub-sample of calves having all test results available did not show any substantial variation and are not shown.

**Table 1:** Descriptive statistics of serum samples used in analyses^1^

Test^2^	n	Mean	SD	First quartile	Third quartile
IgG, g/L	245	16.26	8.91	8.80	22.70
STP_BA, g/L	244	59.21	8.91	53.00	66.00
ALB, g/L	244	22.24	3.21	20.00	24.00
%Brix, %	200	8.83	1.11	8.10	9.60
STP_OP, g/L	241	57.36	10.11	50.00	65.00
GLOB, g/L	244	36.97	7.84	32.00	42.00
GGT activity, IU/L					
All ages	200	561.38	338.90	238.80	952.00
2 and 3 d old	114	640.46	350.31	266.80	1,000.00
4 to 6 d old	86	456.56	293.68	225.80	647.00

1 There were missing values for each test.

2 IgG reference standard was radial immunodiffusion; STP_BA = serum total protein measured by biochemistry analyzer within each veterinary practice; ALB = serum albumin concentration measured by biochemistry analyzer; %Brix = digital Brix refractometer; STP_OP = serum total protein measured by optical refractometer; %Brix = measured by digital refractometer; GLOB = estimated globulin concentration, calculated as STP_BA − ALB; GGT = γ-glutamyl transferase activity measured with biochemistry analyzer.

Results of all the indirect methods were significantly correlated with IgG concentrations as assessed by RID (all *P* < 10^−16^). These correlations were positive and varied from high to very high (r = 0.73, 0.74, 0.87, 0.89, 0.67 for %Brix, STP_OP, STP_BA, GLOB, and GGT activity, respectively). STP_OP and %Brix were also highly correlated (r = 0.78, *P* < 10^−16^), as were STP_OP and STP_BA (r = 0.85, *P* < 10^−16^) and %Brix and STP_BA (r = 0.83, *P* < 10^−16^)

The diagnostic test characteristics of each indirect method for the assessment of FTPI are reported for optimal cutoff values in Table 2. All indirect methods performed globally well (Youden's *J* statistic close to or above 0.7, AUC over 0.80, and d^2^ under 0.25), except STP_OP for which performances were moderate (*J* = 0.51, AUC = 0.80, and d^2^ = 0.35). Following Landis and Koch (1977) guidelines, the agreement between the reference RID assay and STP_OP was “moderate” (κ = 0.52) at the selected optimal cutoff, and “substantial” for all other evaluated tests (κ ranging from 0.63 to 0.76).

**Table 2:** Diagnostic performance of various indirect methods for assessing failure of transfer of passive immunity (FTPI, IgG <10 g/L), compared with radial immunodiffusion as a reference method, at the optimal cutoff points

Test^1^	Optimal cutoff	Sensitivity (95% CI)	Specificity (95% CI)	Statistic^2^			
				*J*	d^2^	AUC	κ
STP_OP, g/L	52.0	69.7 (58.1–79.7)	81.6 (75.0–87.2)	0.51	0.35	0.80	0.52
%Brix, %	8.4	86.5 (74.2–94.4)	83.8 (76.8–89.3)	0.70	0.21	0.88	0.64
STP_BA, g/L	56.0	90.8 (81.9–96.2)	79.2 (72.2–85.0)	0.70	0.22	0.92	0.63
GLOB, g/L	34.0	89.4 (80.3–95.3)	89.3 (83.6–93.5)	0.79	0.15	0.96	0.76
GGT, IU/L							
All ages	254.0	78.8 (65.3–88.9)	90.5 (84.6–94.7)	0.69	0.23	0.90	0.68
2 and 3 d old	393.0	87.5 (71.0–96.5)	87.8 (78.7–94.0)	0.75	0.17	0.92	0.71
4 to 6 d old	254.0	90.0 (68.3–98.8)	86.4 (75.7–93.6)	0.76	0.17	0.91	0.68

1 STP_OP = serum total protein measured by optical refractometer; %Brix = measured by digital refractometer; STP_BA = serum total protein measured by biochemistry analyzer within each veterinary practice; GLOB = estimated globulin concentration, calculated as STP_BA − ALB; ALB = serum albumin concentration measured by biochemistry analyzer; GGT = γ-glutamyl transferase.

2 *J* = Youden's *J* statistic, the maximum of vertical distance of the receiver operating characteristic (ROC) curve from the point (x,y) on the diagonal line (chance line), sensitivity (Se) + specificity (Sp) − 1; d^2^ = square of minimal distance between the point (0, 1) on the upper left-hand corner of ROC space and ROC curve, (1 − Se)^2^ + (1 − Sp)^2^; AUC = area under the ROC curve; κ = Cohen's unweighted kappa statistic.

The highest Se estimates were found for STP_BA (Se = 90.8%, 56 g/L cut-point), GLOB (Se = 89.4%, 34 g/L cut-point), and %Brix (Se = 86.5%, 8.4% cut-point). STP_OP (52 g/L cut-point) was the least sensitive test (Se = 69.7%). For GGT activity, the optimal cut-point varied according to age group and Se estimates were 87.5% for 2- and 3-d-old calves (cut-point 393 IU/L) and 90.0% for 4- to 6-d-old calves (254 IU/L cut-point). Specificity estimates ranged between 79.2% for STP_BA (56 g/L cut-point) and 90.5% for GGT activity (254 IU/L cut-point).

Finally, we report in Table 3 cut-point values for which calves are deemed to suffer or not to suffer from FTPI at the IgG <10.0 g/L threshold, with Sp greater than 95%.

**Table 3:** Cut-point values for whether calves are deemed having or not having failure of transfer of passive immunity (FTPI) with a specificity greater than 95%

Test^1^	Threshold^2^	
	Having FTPI	Not having FTPI
STP_OP, g/L	43.0	68.0
%Brix, %	7.6	9.6
STP_BA, g/L	53.0	61.0
GLOB, g/L	32.0	35.0
GGT, IU/L		
All ages	201.0	583.0
2 and 3 d old	238.0	728.0
4 to 6 d old	164.0	373.0

1 STP_OP = serum total protein measured by optical refractometer; %Brix = measured by digital refractometer; STP_BA = serum total protein measured by biochemistry analyzer with each veterinary practice; GLOB = estimated globulin concentration, calculated as STP_BA − ALB; ALB = serum albumin concentration measured by biochemistry analyzer; GGT = γ-glutamyl transferase.

2 With the best sensitivity when specificity is greater than 95%.

Numerous studies have evaluated the diagnostic accuracy of indirect methods for the assessment of FTPI in calves. In the present study, we estimated the performance of 5 indirect methods applied by veterinary practitioners under field conditions.

The prevalence of FTPI in the study population was high (31.0%) and close to previously reported estimates (Beam et al., 2009; Windeyer et al., 2014; Elsohaby et al., 2015, 2019b; Raboisson et al., 2016). Because calves were selected at random within each herd, this highlighted the fact that FTPI was still frequent in French beef and dairy herds involved in the study. Although the overall sample size was limited, 76 calves were deemed to suffer from FTPI as assessed by the reference RID assay, allowing to get parameter estimates with the desired ±10% absolute precision.

The correlation coefficients of the 2 refractometers compared with the RID determination were similar (r = 0.73 and 0.74) and consistent with other studies using either optical or digital refractometers (Mc Beath et al., 1971; Elsohaby et al., 2015, Hernandez et al., 2016, Cuttance et al., 2017; Elsohaby et al., 2019a), although estimates as high as 0.93 have been reported (Deelen et al., 2014).

For STP_OP and %Brix, the optimal cut-points (52 g/L and 8.4%, respectively) were the same or close to those previously reported for FTPI detection (Tyler et al., 1998; Calloway et al., 2002; Morrill et al., 2013; Deelen et al., 2014; Hernandez et al., 2016; Elsohaby et al., 2019a,b). For STP_OP a higher cut-point (55.0 g/L) has been reported by some authors (Elsohaby et al., 2015). This cut-point has also been suggested in moribund, clinically dehydrated calves (Tyler et al., 1999). Applying this cut-point to our data led to a marginal increase in Se estimate (72.4% *vs* 69.7% at the 52.0 g/L cut-point), but a more important decrease in Sp estimate (74.5% *vs* 81.6%).

The correlation between the 2 refractometers (r = 0.78) was consistent with other studies (Elsohaby et al., 2015) but lower than elsewhere reported (r = 0.91 to 0.97; Hernandez et al., 2016). However, these 2 tests rely on the same principle of measurement. As reported by some veterinarians involved in the present study, these discrepancies may be explained by reading errors or imprecisions on the optical measuring scale, whereas %Brix was found to be easier to read on a digital refractometer. Unfortunately, the intra- or interrater reliability of the evaluated tests were not assessed in the present study, which would have been helpful to investigate this assumption. Instrument variation between the different optical refractometer trademarks might also be invoked to explain these discrepancies (Calloway et al., 2002; Vandeputte et al., 2011; Elsohaby et al., 2015). The high correlation between %Brix and STP (measured by refractometry) found in some studies may be attributed to the use of digital refractometer to determine the STP (Hernandez et al., 2016; Deelen et al., 2014). In the present study, no significant difference was evidenced between the proportion of calves classified as having FTPI by the 2 refractometers (McNemar's test for the Se and Sp comparison, *P*-values = 0.114 and 0.571, respectively), indicating that the 2 refractometers performed similarly for FTPI assessment in calves. The fact that refractometry can be applied to serum harvested without centrifugation from blood samples that have been let to clot (Wallace et al., 2006) reinforces the practicality of STP_OP and %Brix under field conditions.

The assessment of GLOB appeared to have the highest diagnostic performances. This is not surprising, as the possible variations in albumin levels, which influence the STP concentration, were considered (Pfeiffer et al., 1977). Indeed, %Brix, STP_OP, and STP_BA values were all significantly correlated with ALB (r = 0.59, 0.58 and 0.60 respectively, all *P*-values < 10^−16^). Substantial inter-individual variations in ALB were evidenced (Table 1), which may have reflected the hydration status.

Some biological markers, such as STP and globulins concentrations, are highly influenced by dehydration (Fecteau et al., 2013). One advantage of GGT, which is abundant in colostrum and absorbed by the calf intestine (Blum and Hammon, 2000), is its low dependence on hydration status. GGT activity has already been reported to be of informative value for FTPI detection, but it is very sensitive to age (Parish et al., 1997; Šlosárková et al., 2014). In the present study, the mean GGT activity was indeed significantly higher in 2- to 3-d-old calves (640.46 IU/L; SD ± 350.31.20) than in 4- to 6-d-old calves (456.56 IU/L; SD ± 293.68, Wilcoxon signed-rank test: *P*-value <10^−03^). Our optimal cutoffs (Table 2) were consistent with findings in 1- to 7-d-old calves (Perino et al., 1993) and 2- to 8-d-old calves (Cuttance et al., 2017). Lower cut-points have been suggested, however, for d 1 (200 IU/L) or d 4 (100 IU/L) in dairy calves (Parish et al., 1997; Hogan et al., 2015). In contrast to these results, one study was unable to substantiate the utility of serum GGT activity in the prediction of passive transfer status in 69 beef calves younger than 8 d (Wilson et al., 1999). Because of these contradictory findings, studies to determine more accurate thresholds for GGT to assess FTPI depending on calves age are warranted. However, some authors claimed that the use of GGT activity levels should be discouraged, as it offers no advantage over other indirect methods (Weaver et al., 2000).

In almost all studies, including the present one, the assessment of diagnostic test accuracy was made under the assumption that RID is a perfect reference standard. However, imprecision in RID assay replicates from the same sample have been noted (Ameri and Wilkerson, 2008). In the present study, the intra-assay CV of RID for serum samples with IgG expected value around 10.0 g/L was 6.1%. Although intra-assay CV lower than 10% are classically considered acceptable for immunoassays (European Medicines Agency, 2011), the imprecision in RID assay may have led to misclassification of calves actually having or not FTPI at the IgG <10.0 g/L threshold. Recently a Bayesian latent class modeling approach has been proposed to account for the absence of a perfect reference standard (Elsohaby et al., 2019b). Results indicated that RID is not a perfect test, with Se and Sp estimates ranging from 0.92 to 0.96 and 0.91 to 0.93, respectively, depending on the assumptions made for other evaluated tests. This appealing approach requires several populations with different FTPI prevalences and large sample sizes in each herd to yield identifiable statistical models and accurate estimates (Kostoulas et al., 2017). This was not the case for the present study, based on a small number of calves in numerous herds, and this approach could not be applied. However, the influence of imprecision in RID assay could be illustrated by discarding calves with RID-IgG concentration between 9.0 and 11.0 g/L from the analysis. Because optimal cut-points remained unchanged, Se and Sp estimates were all improved (STP_OP: Se = 72.6% and Sp = 84.0%; %Brix: Se = 90.7% and Sp = 87.0%; STP_BA: Se = 92.1% and Sp = 82.9%; GLOB: Se = 93.7% and Sp = 93.0%; GGT: 92.6% and Sp = 90.0% for 2- to 3-d-old calves and Se = 93.8% and Sp = 91.5% for 4- to 6-d-old calves).

Finally, from a practical point of view, farmers or veterinarians are often interested in decision rules associated with a high level of confidence (≥95%) that a calf is suffering or not from FTPI. Comparisons of our suggested decision thresholds (Table 3) with other studies can hardly be made, because such cut-points are seldom reported. However, the suggested cut-points associated with a high level of confidence that a calf is actually suffering from FTPI lie in the range of values reported in a recent meta-analysis (Buczinski et al., 2018) for STP_OP (40–48 g/L) and %Brix (7.6–7.8 g/L). We therefore encourage authors to report such results, as they are of practical interest.

All indirect methods evaluated here can provide reasonably accurate results and be used as effective decision-making tools for determining passive immunity transfer status. From a practicable point of view, however, and despite widespread skepticism and concerns regarding the effects of hydration status, the use of digital refractometer (either measuring STP in g/L, or on the %Brix scale) should be promoted for use FTPI assessment under field conditions.

## References

[bib1] Ameri M., Wilkerson M.J. (2008). Comparison of two commercial radial immunodiffusion assays for detection of bovine immunoglobulin G in newborn calves. J. Vet. Diagn. Invest..

[bib2] Beam A.L., Lombard J.E., Kopral C.A., Garber L.P., Winter A.L., Hicks J.A., Schlater J.L. (2009). Prevalence of failure of passive transfer of immunity in newborn heifer calves and associated management practices on US dairy operations. J. Dairy Sci..

[bib3] Blum J.W., Hammon H. (2000). Colostrum effects on the gastrointestinal tract, and on nutritional, endocrine and metabolic parameters in neonatal calves. Livest. Prod. Sci..

[bib4] Buczinski S., Gicquel E., Fecteau G., Takwoingi Y., Chigerwe M., Vandeweerd J.M. (2018). Systematic review and meta-analysis of diagnostic accuracy of serum refractometry and Brix refractometry for the diagnosis of inadequate transfer of passive immunity in calves. J. Vet. Intern. Med..

[bib5] Calloway C.D., Tyler J.W., Tessman R.K., Hostetler D., Holle J. (2002). Comparison of refractometers and test endpoints in the measurement of serum protein concentration to assess passive transfer status in calves. J. Am. Vet. Med. Assoc..

[bib6] Cuttance E.L., Mason W., Denholm K., Laven R. (2017). Comparison of diagnostic tests for determining the prevalence of failure of passive transfer in New Zealand dairy calves. N. Z. Vet. J..

[bib7] Deelen S.M., Ollivett T.L., Haines D.M., Leslie K.E. (2014). Evaluation of a Brix refractometer to estimate serum immunoglobulin G concentration in neonatal dairy calves. J. Dairy Sci..

[bib8] Donovan G.A., Dohoo I.R., Montgomery D.M., Bennett F.L. (1998). Association between passive immunity and morbidity and mortality in dairy heifers in Florida, USA. Prev. Vet. Med..

[bib9] Elsohaby I., McClure J.T., Keefe G.P. (2015). Evaluation of digital and optical refractometers for assessing failure of transfer of passive immunity in dairy calves. J. Vet. Intern. Med..

[bib10] Elsohaby I., McClure J.T., Waite L.A., Cameron M., Heider L.C., Keefe G.P. (2019). Using serum and plasma samples to assess failure of transfer of passive immunity in dairy calves. J. Dairy Sci..

[bib11] Elsohaby I., Mweu M.M., Mahmmod Y.S., McClure J.T., Keefe G.P. (2019). Diagnostic performance of direct and indirect methods for assessing failure of transfer of passive immunity in dairy calves using latent class analysis. Prev. Vet. Med..

[bib12] European Medicines Agency (2011). Guideline on bioanalytical method validation. https://www.ema.europa.eu/en/documents/scientific-guideline/guideline-bioanalytical-method-validation_en.pdf.

[bib13] Fecteau G., Arsenault J., Paré J., Van Metre D.C., Holmberg C.A., Smith B.P. (2013). Prediction of serum IgG concentration by indirect techniques with adjustment for age and clinical and laboratory covariates in critically ill newborn calves. Can. J. Vet. Res..

[bib14] Godden S. (2008). Colostrum management for dairy calves. Vet. Clin. North Am. Food Anim. Pract..

[bib15] Hajian-Tilaki K. (2013). Receiver operating characteristic (ROC) curve analysis for medical diagnostic test evaluation. Caspian J. Intern. Med..

[bib16] Hernandez D., Nydam D.V., Godden S.M., Bristol L.S., Kryzer A., Ranum J., Schaefer D. (2016). Brix refractometry in serum as a measure of failure of passive transfer compared to measured immunoglobulin G and total protein by refractometry in serum from dairy calves. Vet. J..

[bib17] Hogan I., Doherty M., Fagan J., Kennedy E., Conneely M., Brady P., Ryan C., Lorenz I. (2015). Comparison of rapid laboratory tests for failure of passive transfer in the bovine. Ir. Vet. J..

[bib18] Kostoulas P., Nielsen S.S., Branscum A.J., Johnson W.O., Dendukuri N., Dhand N.K., Toft N., Gardner I.A. (2017). STARD-BLCM: Standards for the reporting of diagnostic accuracy studies that use Bayesian latent class models. Prev. Vet. Med..

[bib19] Landis J.R., Koch G.G. (1977). The measurement of observer agreement for categorical data. Biometrics.

[bib20] Manitz J., Hempelmann M., Kauermann G., Kuechenhoff H., Shao S., Oberhauser C., Westerheide N., Wiesenfarth M. (2017). Samplingbook: Survey sampling procedures. R package version 1.2.2. https://CRAN.R-project.org/package=samplingbook.

[bib21] McBeath D.G., Penhale W.J., Logan E.F. (1971). An examination of the influence of husbandry on the plasma immunoglobulin level of the newborn calf, using a rapid refractometer test for assessing immunoglobulin content. Vet. Rec..

[bib22] Morrill K.M., Polo J., Lago A., Campbell J., Quigley J., Tyler H. (2013). Estimate of serum immunoglobulin G concentration using refractometry with or without caprylic acid fractionation. J. Dairy Sci..

[bib23] Parish S.M., Tyler J.W., Besser T.E., Gay C.C., Krytenberg D. (1997). Prediction of serum IgG1 concentration in Holstein calves using serum gamma glutamyl transferase activity. J. Vet. Intern. Med..

[bib24] Perino L.J., Sutherland R.L., Woollen N.E. (1993). Serum gamma-glutamyltransferase activity and protein concentration at birth and after suckling in calves with adequate and inadequate passive transfer of immunoglobulin G. Am. J. Vet. Res..

[bib25] Pfeiffer N.E., McGuire T.C., Bendel R.B., Weikel J.M. (1977). Quantitation of bovine immunoglobulins: Comparison of single radial immunodiffusion, zinc sulfate turbidity, serum electrophoresis, and refractometer methods. Am. J. Vet. Res..

[bib26] Raboisson D., Trillat P., Cahuzac C. (2016). Failure of passive immune transfer in calves: A meta-analysis on the consequences and assessment of the economic impact. PLoS One.

[bib27] Robin X., Turck N., Hainard A., Tiberti N., Lisacek F., Sanchez J.C., Müller M. (2011). pROC: An open-source package for R and S+ to analyze and compare ROC curves. BMC Bioinformatics.

[bib28] Šlosárková S., Fleischer P., Pěnkava O., Skřivánek M. (2014). The assessment of colostral immunity in dairy calves based on serum biochemical indicators and their relationships. Acta Vet. Brno.

[bib29] Thornhill J.B., Krebs G.L., Petzel C.E. (2015). Evaluation of the Brix refractometer as an on-farm tool for the detection of passive transfer of immunity in dairy calves. Aust. Vet. J..

[bib30] Tyler J.W., Hancock D.D., Wiksie S.E., Holler S.L., Gay J.M., Gay C.C. (1998). Use of serum protein concentration to predict mortality in mixed-source dairy replacement heifers. J. Vet. Intern. Med..

[bib31] Tyler J.W., Parish S.M., Besser T.E., Metre D.C.V., Barrington G.M., Middleton J.R. (1999). Detection of low serum immunoglobulin concentrations in clinically ill calves. J. Vet. Intern. Med..

[bib32] Vandeputte S., Detilleux J., Rollin F. (2011). Comparison of four refractometers for the investigation of the passive transfer in beef calves. J. Vet. Intern. Med..

[bib33] Vogels Z., Chuck G.M., Morton J.M. (2013). Failure of transfer of passive immunity and agammaglobulinaemia in calves in south-west Victorian dairy herds: Prevalence and risk factors. Aust. Vet. J..

[bib34] Wallace M.M., Jarvie B.D., Perkins N.R., Leslie K.E. (2006). A comparison of serum harvesting methods and type of refractometer for determining total solids to estimate failure of passive transfer in calves. Can. Vet. J..

[bib35] Weaver D.M., Tyler J.W., VanMetre D.C., Hostetler D.E., Barrington G.M. (2000). Passive transfer of colostral immunoglobulins in calves. J. Vet. Intern. Med..

[bib36] Wilson L.K., Tyler J.W., Besser T.E., Parish S.M., Gant R. (1999). Prediction of serum IgG_1_ concentration in beef calves based on age and serum gamma-glutamyl-transferase activity. J. Vet. Intern. Med..

[bib37] Windeyer M.C., Leslie K.E., Godden S.M., Hodgins D.C., Lissemore K.D., LeBlanc S.J. (2014). Factors associated with morbidity, mortality, and growth of dairy heifer calves up to 3 months of age. Prev. Vet. Med..

