# Erratum to “A parallel evaluation of 5 indirect cost-effective methods for assessing failure of passive immunity transfer in neonatal calves” (JDS Commun. 1:10–14)

**DOI:** 10.3168/jdsc.2022-3-2-162

**Published:** 2022-03-16

**Authors:** A. Mugnier, K. Pecceu, F. Schelcher, F. Corbiere

The animal ethics statement was missing from this paper. The following statement should be included: “Blood samples were collected as part of the routine calf health management plans conducted by veterinary practitioners on each farm. Ethical approval for animal procedures was deemed not necessary by INRAE-Ecole Nationale Vétérinaire de Toulouse ethical committee.”
